# Tea consumption affects the absorption of levothyroxine

**DOI:** 10.3389/fendo.2022.943775

**Published:** 2022-09-12

**Authors:** Ying-Wen Lai, Shih-Ming Huang

**Affiliations:** Asian Institute of Thyroid Care, Chang Bing Show Chwan Memorial Hospital, Lukang Town, Changhua County, Taiwan

**Keywords:** coffee, levothyroxine, tea, hypothyroidism, thyroid-stimulating hormone

## Abstract

Levothyroxine (LT4) is a convenient treatment for hypothyroidism. Coffee, however, prevents the body from efficaciously absorbing LT4. It is unknown whether the intake of tea interferes with LT4 absorption. Thirty-seven hypothyroidism patients with the following types of consumption were recruited: 1) only tea, 2) tea and coffee, and 3) only coffee. The concentrations of thyroid-stimulating hormone (TSH), triiodothyronine (T3), and thyroxine (T4) were recorded before and 3 months after changing the consumption. The patients had reductions in the concentration of TSH to varying degrees after 3 months (*p* < 0.001 or *p* < 0.05). The natural logarithms of the differences between the concentrations of TSH before and after the change in the consumption (Δln-TSH) of tea and coffee, only coffee, and only tea were 1.94, 2.00, and 2.18, respectively. Long-term consumption of tea, like coffee, interfered with the absorption of LT4. We believe that avoiding tea when taking LT4 will reduce malabsorption.

## Introduction

Levothyroxine (LT4) is the most commonly used drug for the treatment of hypothyroidism in Taiwan. It is often orally administered as a tablet. The gastrointestinal absorption of LT4 is mainly through the jejunum and ileum. Approximately 60%–82% of the administered dose is mostly absorbed during the first 3 h after administration (1). However, before absorption, the dissolution phase is necessary for the tablet form. Physiologic gastric pH is required for dissolution, and gastric disorders or intestinal diseases, such as *Helicobacter pylori* infection, lactose maldigestion, and celiac disease, are known to induce malabsorption of LT4 (2). The interaction between food and LT4 is also well-known due to their physical and chemical properties if they are concomitantly ingested. Coffee significantly affects absorption especially if it is concomitantly ingested with or within 1 h after the drug (3). However, no reports have indicated the interaction between tea and LT4.

In Taiwan, the consumption of coffee has increased rapidly in recent years, while due to the wide consumption of tea, detailed history has shown that drinking tea soon after taking LT4 was associated with its malabsorption. The possibility of similar effects of coffee and tea has been considered because of their mutual compounds. Because no report had indicated the interaction between tea and LT4, it was important to determine whether this was significant or not, particularly in Asian countries such as Taiwan, Japan, and Korea. A prospective cohort study was therefore conducted to uncover the association between the consumption of tea and the malabsorption of LT4.

## Patients and methods

### Patient population and data collection

All the recruited patients were regular visitors of Dr. Shih-Ming Huang’s Outpatients Department due to the follow-up of thyroid disease. They received medical treatment, and their thyroid functions were well controlled in recent years. The LT4 dose and thyroid function in the past years had been steady and euthyroidism or subclinical hyperthyroidism had been maintained. The concentration of the TSH of the patients with thyroid cancer was maintained at <1.00 mIU/L, and that of the patients with benign disease was maintained at <3.00 mIU/L. However, those patients had suspicious malabsorption of LT4, and their histories for patterns similar to the following were investigated (1): the patients swallowed the LT4 tablets with water, not with coffee or tea, but started consuming tea and/or coffee within 1 h of LT4 intake within the most recent half-year; (2) the serum thyroid-stimulating hormone (TSH) appeared abnormally higher than the target; and (3) the patients did not have any gastric or intestinal disorders nor any antacid medicine, soybean milk, and vitamin C intake. In Taiwan, tea is available from supermarkets or homemade; the preparation processes are similar for both: people just drink the liquid part and do not eat the leaves. Coffee is consumed in two main ways: black coffee or coffee mixed with milk, but very rarely consumed as mocha or pure espresso. The patients were asked to keep the same dose of LT4 but avoid coffee and tea at least 4 h before and after LT4 intake. The TSH, T3, and T4 concentrations after changing consumption for 3 months were recorded. The patients were divided into three groups, based on what they consumed: only tea, only coffee, and both tea and coffee. Other subgroups were based on age and gender, such as “those aged above 60”, “male”, and “female”.

### Statistical analysis

SPSS statistics version 17.0 (IBM Corp.) was used for statistical analysis. The significance of the difference between the pre- and post-TSH concentrations was calculated using the paired *t*-test. The pre-TSH concentration referred to the first recorded abnormal serum TSH concentration. On the other hand, the post-TSH concentration was the concentration of TSH 3 months after pre-TSH during follow-up. Δln-TSH, which is defined as the difference between the natural logarithms of the pre- and post-TSH concentrations, was used for analysis. We used the natural logarithm to express the difference because the minimum post-TSH concentration was 0.001; however, the maximum pre-TSH concentration was 35.84 mIU/L. The significance of the differences between the pre-T3, pre-T4, post-T3, and post-T4 concentrations were also determined using the paired *t*-test. Variables of different groups were analyzed by one-way ANOVA. The Scheffe test, as a post-hoc analytical test, was used for multiple comparison. A *p-*value of <0.05 was considered to denote statistical significance.

## Results

In the Huang clinics, approximately 2,000 patients with primary hypothyroidism, thyroid cancer, and Graves’ disease after total thyroidectomy with or without radioiodine treatment had been regularly followed up on every 6 months. Approximately 70 cases with hypothyroidism were found during the first 6 months of 2017, but only 37 cases met the inclusion criteria. A total of 37 patients were included in this study, of which 13 drank only coffee within 1 h of LT4 intake, 18 patients drank only tea within 1 h of LT4 intake, and six patients drank both coffee and tea within 1 h of LT4 intake. There were no statistically significant differences in the demographic data including sex, age, underlying gastrointestinal diseases, weekly T4 dose, and the prevalence of Graves’ disease or cancer (**Table 1**).

**Table 1:** Demographic data of the participants.

	Only coffee			Only tea			Both coffee and tea			
	N	%		N	%		N	%		*P*-value
All	13			18			6			
Sex										0.544
Female	10	76.9		16	88.9		6	100.0		
Male	3	23.1		2	11.1		0	0.0		
Age, year										
Mean, SD (Range)	51.9	15.4	(20–80)	47.6	15.2	(26–75)	55.2	12.4	(37–73)	0.446
Weekly T4 dose (μg)										
Mean, SD (Range)	1,007.7*	301.3	(400–1,500)	791.7*	175.1	(500–1,200)	900.0	141.4	(700–1,100)	0.027
Disease										0.899
Graves’ disease	5	38.5		6	33.3		3	50.0		
Cancer	8	61.5		12	66.7		3	50.0		

P-value by Fisher’s exact test or Kruskal-Wallis test. *: P < 0.05 by Tukey multiple comparison analysis.

**Figure 1:**
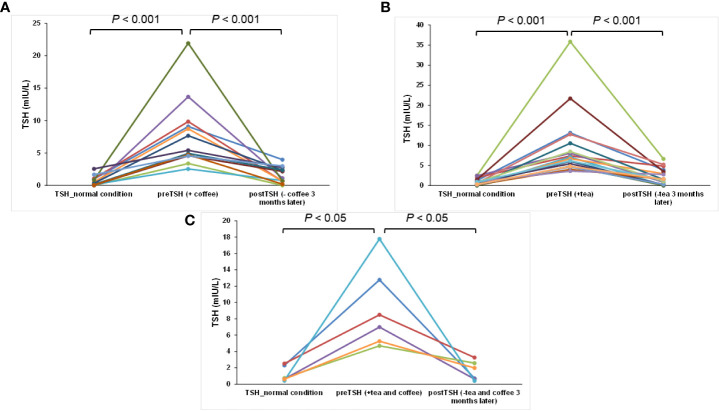
The time course for TSH changes for each patient for normal conditions, regular tea and/or coffee consumption within 1 h of LT4 intake, and coffee/tea consumption at least 4 h after swallowing LT4: **(A)** only coffee, **(B)** only tea, and **(C)** both coffee and tea groups.

**Figure 2:**
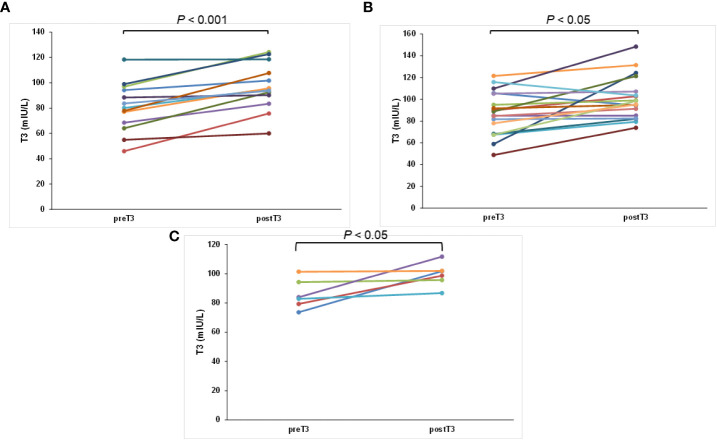
The change in T3 concentration for each patient after swallowing the tablet with water and postponing coffee/tea at least 4 h later: **(A)** only coffee, **(B)** only tea, and **(C)** both coffee and tea groups.

**Figure 3:**
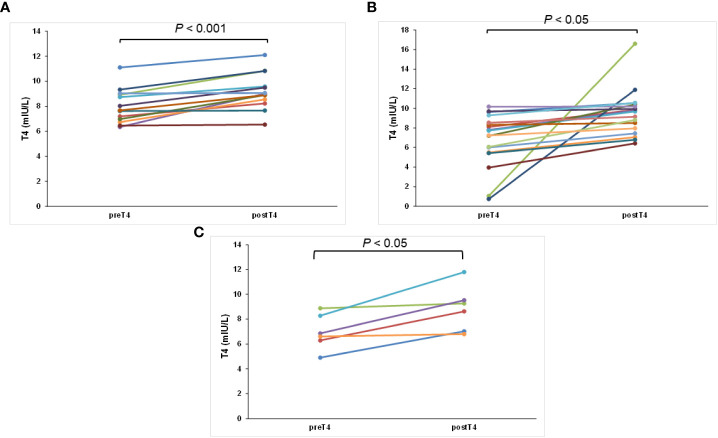
The change in T4 concentration for each patient after swallowing the tablet with water and postponing coffee/tea consumption at least 4 h later: **(A)** only coffee, **(B)** only tea, and **(C)** both coffee and tea groups.

**Figure 1** shows the differences between the pre- and post-TSH concentrations of the three groups. After 3 months without tea and/or coffee consumption within 1 h of LT4 intake, all 37 patients had varied reductions in the concentrations of TSH. The post-TSH was significantly less than the pre-TSH concentration for all the three groups (*p* < 0.001, < 0.001, and < 0.05, respectively, **Figure 1**). The maximum pre-TSH concentration was 35.84 mIU/L; this pre-TSH concentration decreased to 6.62 mIU/L. The minimum pre-TSH concentration was 2.54 mIU/L, and it decreased to 0.75 mIU/L 3 months later. These results indicate that the consumption of only coffee, only tea, and both coffee and tea interfered with the absorption of levothyroxine significantly. Furthermore, the serum post-T3 and post-T4 concentrations increased from the pre-T3 and pre-T4 with statistically significant differences according to the paired *t*-test for all three groups, indicating better results caused by better absorption of LT4 (both *p* < 0.001, *p* < 0.05, *p* < 0.05, respectively, **Figures 2**, **3**).

**Tables 2**, **3** show the mean Δln-TSH. The Δln-TSH of the patient who drank tea and coffee concomitantly was 1.94 ± 1.28. The Δln-TSH values of the patients drinking only coffee and only tea were 2.00 ± 1.56 and 2.18 ± 1.72, respectively. There were no statistically significant differences among the three groups.

**Table 2:** Comparison of the pre- and post-TSH concentrations and ΔlnTSH of the patients in the only tea, only coffee, and both coffee and tea groups.

	Only coffee (N = 13)			Only tea (N = 18)			Both coffee and tea^¶^ (N = 6)			
	Mean	SD	Range	Mean	SD	Range	Mean	SD	Range	*P*-value^a^
Pre-TSH^†^ (mIU/L)	7.78	5.26	(2.54–21.93)	9.51	7.90	(3.61–35.84)	9.33	5.05	(4.69–17.78)	0.652
Post-TSH^‡^ (mIU/L)	1.59	1.24	(0.01–3.98)	2.13	1.99	(0.01–6.62)	1.59	1.18	(0.41–3.26)	0.861
Pre-post test *P*-value^b^	0.001			<0.001			0.028			
ΔlnTSH^§^	2.00	1.56	(0.42–5.82)	2.18	1.72	(0.27–6.40)	1.94	1.28	(0.60–3.77)	0.958

**^†^** The first time we recorded abnormally elevated TSH concentrations.

**^‡^** The patients were asked to change their concomitant consumption of coffee or tea with the drug. We recorded the serum TSH concentration 3 months after the pre-TSH concentration was recorded during follow-up.

^§^ΔlnTSH = the difference between the natural logarithms of the pre- and post-TSH concentrations.

^¶^Both coffee and tea: The patients had the habit of drinking tea and coffee concomitantly.

a P-value by Kruskal-Wallis test for the difference among groups.

b P-value by Wilcoxon signed-rank test for pre- and post-TSH concentrations comparison within group.

**Table 3:** Comparison of the pre- and post-TSH concentrations and ΔlnTSH of the different groups stratified by sex and age.

		Pre-TSH^†^ (mIU/L)			Post-TSH^‡^ (mIU/L)				ΔlnTSH^§^		
	N	Mean	SD	Range	Mean	SD	Range	*P*-value^b^	Mean	SD	Range
Sex											
Female	32	8.68	6.57	(2.54–35.84)	1.70	1.67	(0.01–6.62)	<0.001	2.22	1.62	(0.27–6.40)
Male	5	10.09	7.02	(4.55–21.69)	2.82	1.00	(1.49–3.98)	0.043	1.16	0.69	(0.42–1.96)
*P*-value^a^		0.780			0.057				0.117		
Age, year											
<60	25	9.58	7.12	(3.38–35.84)	1.76	1.77	(0.01–6.62)	<0.001	2.41	1.71	(0.42–6.40)
≥60	12	7.40	5.14	(2.54–21.69)	2.06	1.33	(0.20–4.83)	0.002	1.39	0.95	(0.27–3.17)
*P*-value^a^		0.240			0.360				0.066		

**^†^** The first time we recorded abnormally elevated TSH concentrations.

**^‡^** The patients were asked to change their concomitant consumption of coffee or tea with the drug. We recorded the serum TSH concentration 3 months after the pre-TSH concentration was recorded during follow-up.

^§^ΔlnTSH = the difference between the natural logarithms of the pre- and post-TSH concentrations.

a P-value by Mann-Whitney U test for the difference between groups.

b P-value by Wilcoxon signed-rank test for pre and post-TSH concentrations comparison within group.

The Δln-TSH of the female patients was 2.22 ± 1.62, and that of the male patients was 1.16 ± 0.69. The Δln-TSH of the patients older than 60 years was 1.39 ± 0.95. The Δln-TSH of the patients younger than 60 years was 2.41 ± 1.71. There were no statistically significant differences related to sex and age (**Table 3**).

## Discussion

Tea has been part of Taiwan’s culture for a long time because it is also an element of Chinese culture. On the other hand, coffee consumption seems to have rapidly increased recently with a varied selection of coffee products (4) being made available. This is consistent with our clinical experience; more cases had unstable concentrations of TSH that could be related to coffee consumption, and most of these cases normalized after the cessation of coffee consumption.

When patients drank coffee or tea within 1 h of taking drugs, it was confirmed to affect the absorption of LT4 (3). In the Huang clinics, approximately 2000 patients had primary hypothyroidism; 13 and 18 patients (0.65% and 0.9%) had consumed coffee and tea, respectively. In the review by Benvenga (5), 4 of 210 adult patients taking LT4 therapy had impaired LT4 absorption caused by coffee intake (1.9%). Thus, our ratio of patients with impaired LT4 absorption caused by coffee was lower than that reported by Benvenga. This may indicate that Asian people drink more tea, and tea consumption becomes a more significant issue related to the malabsorption of LT4. In addition, sex and age may not have been the main factors related to malabsorption. LT4 sodium is absorbed along the entire small intestine at different rates, but it stays in the stomach long before absorption. In the previous study, it was shown that LT4 sodium stayed in the stomach for 35 ± 30 min, in the duodenum for 7 ± 3 min, and in the upper jejunoileum for 31 ± 8 min (6). Therefore, the main factors affecting these processes are gastric juice pH and viscosity, type of excipients and structure, and shape and dimension of active ingredient particles (2). Corresponding to fasting, the peak value of LT4 absorption after a meal is decreased, and Tmax (the amount of time to reach the maximum concentration) is delayed, with a resulting decrease in LT4 bioavailability. Certain foods and drinks were recorded to be associated with a decrease in the absorption of LT4, such as soybeans, coffee, and papaya (2). Additionally, former studies have indicated that bedtime intake of T4 significantly improves thyroid hormone efficacy due to increased exposure of T4 to the intestinal mucosa at night (6) or better patient compliance with the treatment (7). Coffee may lower the absorption of both inorganic and organic compounds, since it was confirmed as a weak sequestrant of LT4 in an *in vitro* study (3). Because tea and coffee share many mutual components, such as caffeine, polysaccharides, and polyphenols, we believe tea also lowers the absorption of LT4. In the former study, polyphenol was confirmed to decrease the absorption of protein and lipid in the intestine (8). Catechin, one of the well-known polyphenols, showed inhibitory effects on glucose absorption in intestinal cells due to the competitive inhibition of Na-glucose co-transporter 1 (9).

The tablet form of LT4, which has been used for a long time, was malabsorbed with several foods and coffee, proton pump inhibitors (PPIs), gastritis, and bariatric surgery (10). Changes to different products, brands, or formulations may also cause health issues (11). Therefore, different forms of LT4 were developed, particularly a liquid solution and soft capsule (12). Several studies have reported that a liquid solution or capsule was more effective than a tablet or placebo when there were factors related to malabsorption. These studies usually lasted for months to observe long-term outcomes (13, 14), and they provided insights into the influence of malabsorption on TSH.

Although this cohort study had a relatively small sample size, the results provide obvious and valuable insights. We believe that tea, as well as coffee, reduces the absorption of LT4. Therefore, tea should be added to the list of substances that cause LT4 malabsorption. Although most Taiwanese already tend to avoid having coffee or tea at night, we suggest that patients in Taiwan take LT4 during bedtime to prevent avoidable malabsorption.

## Data availability statement

The original contributions presented in the study are included in the article/supplementary material. Further inquiries can be directed to the corresponding author.

## Ethics statement

The studies involving human participants were reviewed and approved by Show Chwan Memorial Hospital. Written informed consent for participation was not required for this study in accordance with the national legislation and the institutional requirements.

## Author contributions

Y-WL: Data acquisition and analysis and writing of the article. S-MH: Study design, data interpretation, editing of the article and funding acquisition. Both authors contributed to the article and approved the submitted version.

## Funding

The authors gratefully acknowledge the Summer Research Project Grant no. NCKUMCS2017040 from College of Medicine at National Cheng Kung University.

## Acknowledgments

Y-WL expresses special thanks to colleagues Dr. Hsin-Yu Huang and Dr. Hao-Chun Chuang who gave the author precious suggestions.

## Conflict of interest

The authors declare that the research was conducted in the absence of any commercial or financial relationships that could be construed as a potential conflict of interest.

## Publisher’s note

All claims expressed in this article are solely those of the authors and do not necessarily represent those of their affiliated organizations, or those of the publisher, the editors and the reviewers. Any product that may be evaluated in this article, or claim that may be made by its manufacturer, is not guaranteed or endorsed by the publisher.
